# Relationship between short-term exposure to sulfur dioxide and emergency ambulance dispatches due to cardiovascular disease

**DOI:** 10.1097/EE9.0000000000000341

**Published:** 2024-09-24

**Authors:** Xuerui Bai, Hongying Qu, Zebing Ye, Ruoting Wang, Guanhao He, Zhongguo Huang, Zhiying Jiang, Changfa Zhang, Shuai Li, Guowei Li

**Affiliations:** a Center for Clinical Epidemiology and Methodology (CCEM), The Affiliated Guangdong Second Provincial General Hospital of Jinan University, Guangzhou, China; b Department of Public Health and Preventive Medicine, School of Medicine, Jinan University, Guangzhou, China; c Department of Cardiology, The Affiliated Guangdong Second Provincial General Hospital of Jinan University, Guangzhou, China; d Father Sean O’Sullivan Research Centre, St Joseph’s Healthcare Hamilton, Hamilton, Ontario, Canada

**Keywords:** CVD, SO_2_, Distributed lag nonlinear model, Emergency ambulance dispatches

## Abstract

**Background:**

The relationship between sulfur dioxide (SO_2_) and cardiovascular disease (CVD) remains inconclusive. We aimed to clarify the association between short-term exposure to SO_2_ and emergency ambulance dispatches (EADs) due to CVD.

**Methods:**

We collected daily data on the number of EADs due to CVD, air pollutants, and meteorological factors between October 2013 and June 2018 in Guangzhou, China. We used the quasi-Poisson generalized additive model combined with a distributed lag nonlinear model to estimate the short-term effect of SO_2_ on EADs due to CVD in multivariable models. Subgroup and sensitivity analyses were also performed.

**Results:**

A total of 37,889 EADs due to CVD were documented during the study period. The average daily SO_2_ concentration was 12.5 μg/m^3^. A significant relationship between SO_2_ and EADs due to CVD was found, with a relative risk of 1.04 (95% confidence interval: 1.02, 1.06) with each 10 μg/m^3^ increment of SO_2_ at lag 0-1_._ The relationship was stronger in males, for participants aged ≥65 years, and in the cold season; however, no significant modification by subgroup was found in the association between SO_2_ and EADs due to CVD. Similar results from sensitivity analyses to the main findings were observed.

**Conclusions:**

Short-term exposure to SO_2_ was significantly associated with increased EADs due to CVD.

**What this study adds**

Cardiovascular disease (CVD) is the leading cause of mortality worldwide, while the relationship between sulfur dioxide (SO_2_) and CVD remains inconclusive. We assessed the association between exposure to SO_2_ and emergency ambulance dispatches (EADs) due to CVD in Guangzhou, China. It was found that short-term exposure to SO_2_ was significantly associated with EADs due to CVD. The relationship between SO_2_ and EADs due to CVD was stronger in males, for participants aged ≥65 years, and in the cold season.

## Introduction

Cardiovascular disease (CVD) remains the leading cause of mortality worldwide.^1^ In 2020, CVD caused approximately 19 million deaths globally, which amounted to an increase of 18.7% from 2010.^2^ It was well documented that exposure to air pollutants exerted adverse effects on CVD.^3,4^ For instance, prior animal studies confirmed that sulfur dioxide (SO_2_), a harmful air pollutant, could induce undue inflammatory responses and oxidative damages to increase the risk of CVD.^5^

SO_2_ is emitted primarily by the combustion of fossil fuels, power plants, and other industrial processes.^6^ Rapid industrialization-driven economic growth is coupled with increased SO_2_ emission in China, in which China has become the largest emitter worldwide.^7,8^ While there was growing evidence of ambient air pollutants in relation to CVD,^9,10^ the relationship between SO_2_ and CVD remained inconsistent from population studies. Some studies in China reported that SO_2_ was significantly associated with an increased risk of CVD.^4,11^ By contrast, several studies found no significant association between SO_2_ and CVD.^12,13^ For example, a case-crossover analysis conducted in England and Wales found a negative association that was not statistically significant.^12^ More evidence is therefore needed to further clarify the relationship between SO_2_ and CVD.

It was reported that health databases, including hospital admission data, mortality data, and electronic medical records, may lack the sensitivity to detect acute health events.^14,15^ By contrast, emergency ambulance dispatches (EADs) were recognized as a sensitive indicator for capturing acute health events and reflecting the demands for medical resources among communities.^16^ When participants called ambulances for acute and life-threatening conditions, rapid transport along with initial diagnosis and treatment in ambulances was crucial for life-saving and participants’ outcomes.^17,18^ EADs would be more appropriate to explore the short-term exposure to SO_2_ in relation to CVD risk from the perspective of public health policy making when compared with using data from participants’ electronic medical records.^14^ Thus, in this study we aimed to assess the association between short-term exposure to SO_2_ and EADs due to CVD, based on data from Guangzhou, China between 2013 and 2018. Study findings may help clarify the relationship between SO_2_ and EADs due to CVD and thus help adequately optimize the medical resource allocation for healthcare centers and policy makers.

## Methods

### Study setting

Guangzhou as the capital of Guangdong Province, is an important economic center located in Southern China with an area of 7434.4 km^2^ and a population of 15.3 million (as of 2021).^16,19^ Guangzhou owns a subtropical humid-monsoon climate, where the average annual temperature is 22 °C and the average rainfall is 1500–2000 mm.^20^ The economic development of Guangzhou is accompanied by serious air pollutants. Although the Guangzhou government has implemented control measures to improve the air quality, the air pollutants situation remains serious.^21^ This study received an ethical waiver because all data were publicly accessible and patient privacy was not involved.

### Outcome measures

Our primary outcome was EADs due to CVD, and secondary outcomes included EADs due to stroke, myocardial infarction (MI), heart failure (HF), and arrhythmia. Daily EADs were extracted from the Guangzhou Emergency Center, where the data were available from 28 October 28 2013 to 30 June 2018 for our study.

The Emergency Center established a dynamic dispatching system in the urban area of Guangzhou, owning over 200 ambulances equipped with resuscitative equipment to provide emergency medical services for about seven million citizens.^22^ The center ensured that an ambulance arrived at the scene within 30 minutes after emergency calls even during the night.^23,24^ Accuracy of the data on participants’ information and EADs due to CVD had been justified in previous publications.^16,20^ Information related to each case was recorded by trained medical physicians, including the ambulance dispatch time, age, sex, district, symptoms, and preliminary diagnosis. The diagnosis of CVD was obtained by field emergency physicians based on patients’ symptoms, inquiries, and physical examination.^16^ All physicians applied standardized procedures to ensure the accuracy and completeness of information.

### Air pollutants and meteorological data

Data on the daily air pollutants were obtained from the Guangzhou Bureau of Environmental Protection (https://sthjj.gz.gov.cn/). There were 11 fixed-site air pollutant monitoring stations in Guangzhou providing daily concentration for each pollutant, including 24-hour average concentrations of SO_2_, particulate matter less than 2.5 μm in aerodynamic diameter (PM_2.5_), particulate matter less than 10 μm in aerodynamic diameter (PM_10_), nitrogen dioxide (NO_2_), carbon monoxide (CO), and the maximum 8-hour moving average of ozone (O_3_). The daily average concentrations of pollutants from all monitoring stations were calculated to obtain daily air pollutant concentrations at the city level in Guangzhou.^25^ More than half of the stations were located in the central urban area of Guangzhou. SFigure 1; http://links.lww.com/EE/A301 shows the geographical location of 11 air monitoring stations in Guangzhou.

Daily meteorological data during the study period were collected from Guangzhou Weather Station (http://www.tqyb.com.cn/), in which the meteorological information included minimum temperature, maximum temperature, and relative humidity. We calculated the average of minimum and maximum temperature as the daily mean temperature.^26^ During the study period, there were no missing data on air pollutants and meteorology.

### Statistical analysis

Descriptive analysis was performed for EADs, air pollutants, and meteorological factors by using mean, standard deviation (SD), minimum (Min), 25th percentile (P25), 50th percentile (P50), 75th percentile (P75), and maximum (Max). Additionally, daily numbers of EADs due to CVD, stroke, MI, HF, and arrhythmia were plotted as time series to present potential temporal trends.

In order to avoid overdispersion of EADs data, we used the quasi-Poisson generalized additive model combined with a distributed lagged nonlinear model (DLNM) to estimate the relationship between SO_2_ and EADs due to CVD, after adjusting for long-term trend, mean temperature and relative humidity, day of week, and public holidays. The model is shown as follows:

Log[E(*Y*_*t*_)] = 𝛼 + 𝛽𝑋_𝑡,𝑙_ + S(Time*, 𝑑𝑓 =* 6/year) + S(Temp, *𝑑𝑓 =* 3) + S(RH, *𝑑𝑓 =* 3) + *γ*DOW *+ δ*Holiday

where E(*Y*_*t*_) means the expected count of EADs at day *t; α* is the model’s intercept; 𝑋_𝑡,𝑙_ produced by DLNM is the cross-basis matrixes of SO_2_ concentration at day 𝑡 and lag *l*, and *β* is the coefficient of the matrix; S(Time, *𝑑𝑓*) denotes the smoothing spline function to control the long-term trend and seasonality, which is the sequence from day 1 to 1707 in this study; S(Temp, *𝑑𝑓*) and S(RH, 𝑑*f*) are the smoothing spline functions to control daily mean temperature and daily relative humidity, respectively; *df* is the degree of freedom; DOW refers to the indicator variable for the day of the week; Holiday is the variable for the public holidays effect; *γ* and *δ* are coefficients.

The quasi-Akaike information criterion (Q-AIC) was used to determine the *df*, with a smaller Q-AIC indicating a better fit for the model.^27^ Based on the Q-AIC measures (STable 1; http://links.lww.com/EE/A301), we applied 6 *df* per year for time trend and 3 *df* for average temperature and relative humidity. These choices of *df* were consistent with previous studies.^28,29^ Given that previous studies consistently reported the strongest effect estimates at lag 0-1,^7,30^ in this study we assessed the cumulative lag effect of SO_2_ at lag 0-1 as our primary analysis. To estimate the overall associations between the two-day moving average (i.e., lag 0-1) of SO_2_ and EADs due to CVD, we first conducted a linearity test and found a linear concentration–response relationship (*P*_for nonlinearity_ = 0.36). Thus, we selected a natural cubic spline function with 3 *df* for lag-response and a linear function for exposure–response to plot the concentration–response curve.^31^ Similarly, SO_2_ was incorporated into the model to explore the potential delayed influence on EADs due to stroke, MI, HF, and arrhythmia.

The effects of SO_2_ on EADs at different lag days were explored as a supplementary analysis, including single-day lags (lag 0 to lag 5) and cumulative-day lags (lag 0-1 to lag 0-5) because the effects of SO_2_ could persist up to 5 days according to previous findings.^32,33^ We also performed subgroup analyses by sex (males vs. females), age (<65 years vs. ≥65 years), and season (cold vs. warm) to explore potential effect modifications of the association between SO_2_ and EADs due to CVD. In Guangzhou, the cold season was from October to March, while the warm season was from April to September.

To evaluate the robustness of results from our primary analysis, we conducted two sensitivity analyses. First, we assessed the relationship between SO_2_ and EADs due to CVD after further adjusting for other air pollutants including PM_2.5_, PM_10_, O_3_, NO_2_, and CO. We used Spearman correlation test to evaluate the correlation between these air pollutants before checking their variance inflation factors (VIF) to enter the model. All air pollutants had a VIF < 10, indicating no severe multicollinearity. Therefore, these other air pollutants were all further adjusted for in the multivariable model. Second, we further adjusted for participants’ sex and age in the model as another sensitivity analysis. The daily percentages for age (i.e., the percentage of participants aged <65 years old) and sex (i.e., the percentage of females) were used to adjust for the potential effects of age and sex in the model on the association between SO_2_ and EADs due to CVD.^16^

The relationship between SO_2_ and EADs due to CVD was quantified as relative risk (RR) and 95% confidence intervals (CIs) with each 10 μg/m^3^ increase in SO_2_. All statistical analyses were performed in R version 4.2.3 (R Core Team 2023, Vienna, Austria) with “dlnm” and “mgcv” packages. A *P* value <0.05 was considered statistically significant.

## Results

During the study period, there were a total of 37,889 EADs due to CVD, among which there were 13,196 stroke events, 2356 MI events, 3366 HF events, 4023 arrhythmia events, and 14,948 other CVD events (Table 1). About 74.1% of EADs cases occurred in the central urban area of Guangzhou. The daily concentration of SO_2_ had an average of 12.5 (SD = 6.0) μg/m^3^ (SFigure 2; http://links.lww.com/EE/A301 shows the distribution of SO_2_ concentration). Notably, 58.5% of the participants were males and 63.3% aged ≥65 years. The average temperature and relative humidity were 22.9 °C and 76.1%, respectively.

**Table 1.** Description of air pollutants, meteorological factors, and EADs between October 2013 and June 2018 in Guangzhou, China

	Mean	SD	Min	P25	P50	P75	Max
Air pollutants							
Daily SO_2_ concentration (μg/m^3^)	12.5	6.0	2.0	8.0	11.0	15.0	53.0
Daily PM_2.5_ concentration (μg/m^3^)	39.3	23.0	4.0	23.0	34.0	50.0	155.0
Daily PM_10_ concentration (μg/m^3^)	60.4	30.2	9.0	38.0	53.0	76.0	208.0
Daily NO_2_ concentration (μg/m^3^)	46.2	19.2	13.0	33.0	42.0	55.0	163.0
Daily O_3_ concentration (μg/m^3^)	46.4	25.1	3.0	26.0	43.0	62.0	139.0
Daily CO concentration (mg/m^3^)	0.9	0.2	0.5	0.8	0.9	1.1	2.6
Meteorological factors							
Daily temperature (°C)	22.9	6.5	3.0	18.0	24.0	28.5	32.5
Daily relative humidity (%)	76.1	15.0	15.0	67.0	80.0	88.0	97.0
Daily number of EADs							
By cause							
* *EADs due to CVD	22.3	6.0	6.0	18.0	22.0	26.0	59.0
* *EADs due to stroke	7.8	3.1	0.0	6.0	8.0	10.0	22.0
* *EADs due to MI	1.4	1.3	0.0	0.0	1.0	2.0	9.0
* *EADs due to HF	2.0	1.6	0.0	1.0	2.0	3.0	10.0
* *EADs due to arrhythmia	2.4	1.7	0.0	1.0	2.0	3.0	10.0
* *EADs due to other CVDs^a^	8.8	3.6	0.0	6.0	8.0	11.0	45.0
By sex^b^							
For male participants	11.5	4.0	2.0	9.0	11.0	14.0	27.0
For female participants	8.2	3.4	0.0	6.0	8.0	10.0	25.0
By age^c^							
* *For participants <65 years	7.7	3.1	1.0	6.0	7.0	10.0	43.0
* *For participants ≥65 years	13.3	4.6	2.0	10.0	13.0	16.0	35.0
By season^d^							
* *In warm season	20.6	5.3	6.0	17.0	20.0	24.0	59.0
* * In cold season	24.0	6.1	9.0	20.0	24.0	28.0	53.0

a Other CVDs included cardiac arrest and angina.

b There were 4507 missing values in sex.

c There were 2294 missing values in age.

d The warm season was from April to September, and the cold season was from October to March.

Max indicates maximum; Min, minimum; P25, 25th percentile; P50, 50th percentile; P75, 75th percentile.

The numbers of daily EADs due to CVD and secondary outcomes by the study period are shown in SFigure 3; http://links.lww.com/EE/A301. Fluctuating trends were observed in the daily numbers of EADs. The average daily numbers of EADs due to CVD, stroke, MI, HF, and arrhythmia were 22.3 (SD = 6.0), 7.8 (SD = 3.1), 1.4 (SD = 1.3), 2.0 (SD = 1.6) and 2.4 (SD = 1.7), respectively (Table 1).

**Figure 1.**
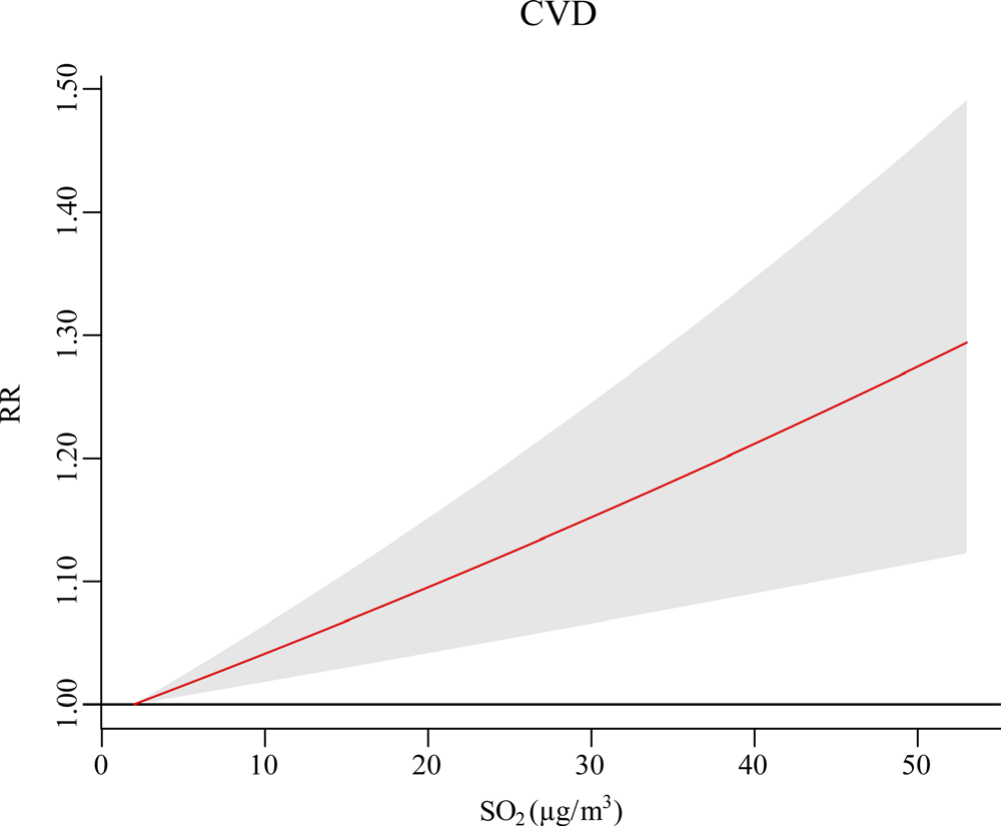
The concentration–response curve between SO_2_ and EADs due to CVD at lag 0-1.

**Figure 2.**
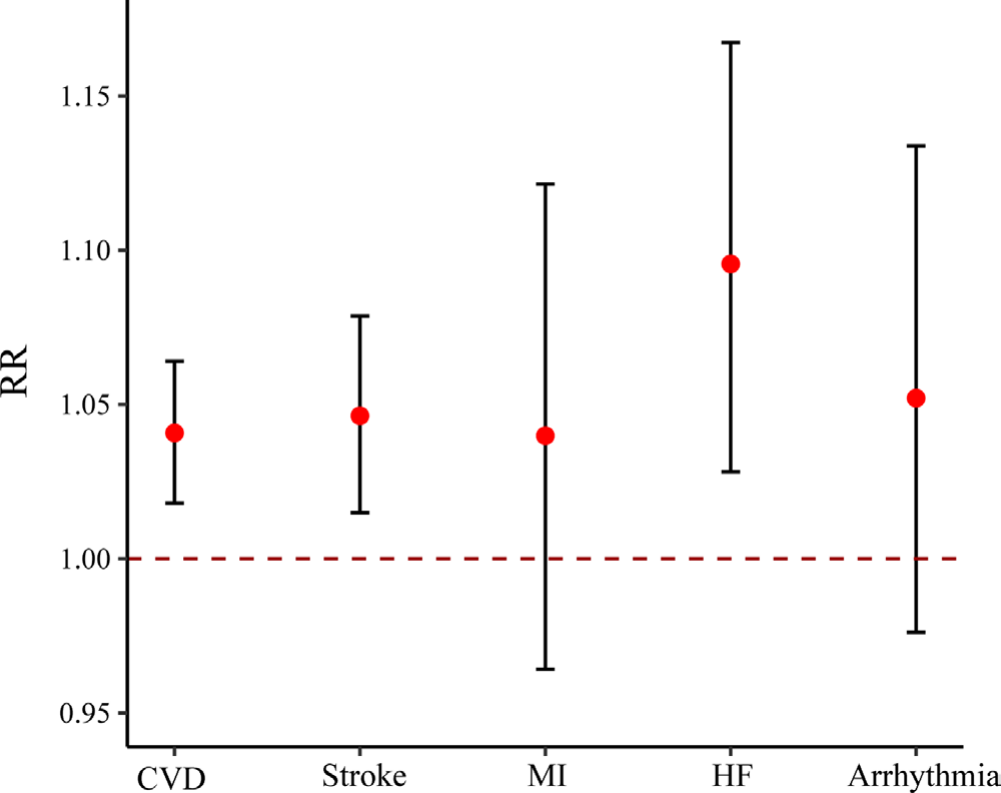
Results for the relationship between each 10 μg/m^3^ increase in SO_2_ and EADs due to CVD, stroke, MI, HF, and arrhythmia at lag 0-1.

**Figure 3.**
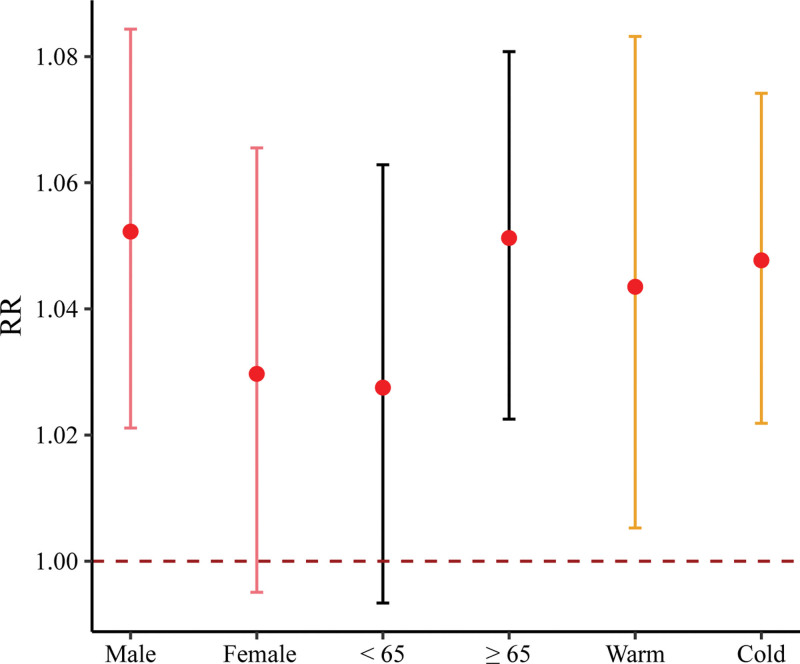
Results for the relationship between each 10 μg/m^3^ increase in SO_2_ and EADs due to CVD among different subgroups at lag 0-1.

Figure 1 displays the linear concentration–response curve between SO_2_ and risks of EADs due to CVD at lag 0-1. As shown in Figure 2, a significant association between SO_2_ and EADs due to CVD was found at lag 0-1 (RR = 1.04, 95% CI: 1.02, 1.06 with each 10 μg/m^3^ increase in SO_2_)_._ When compared with females and participants aged <65 years, respectively, the relationship between SO_2_ on EADs due to CVD was stronger in males and for those aged ≥65 years (Figure 3). A larger RR of EADs due to CVD was also found in cold season than warm season. Nevertheless, no significant modification by these subgroups was found in the association between SO_2_ and EADs due to CVD.

**Figure 4.**
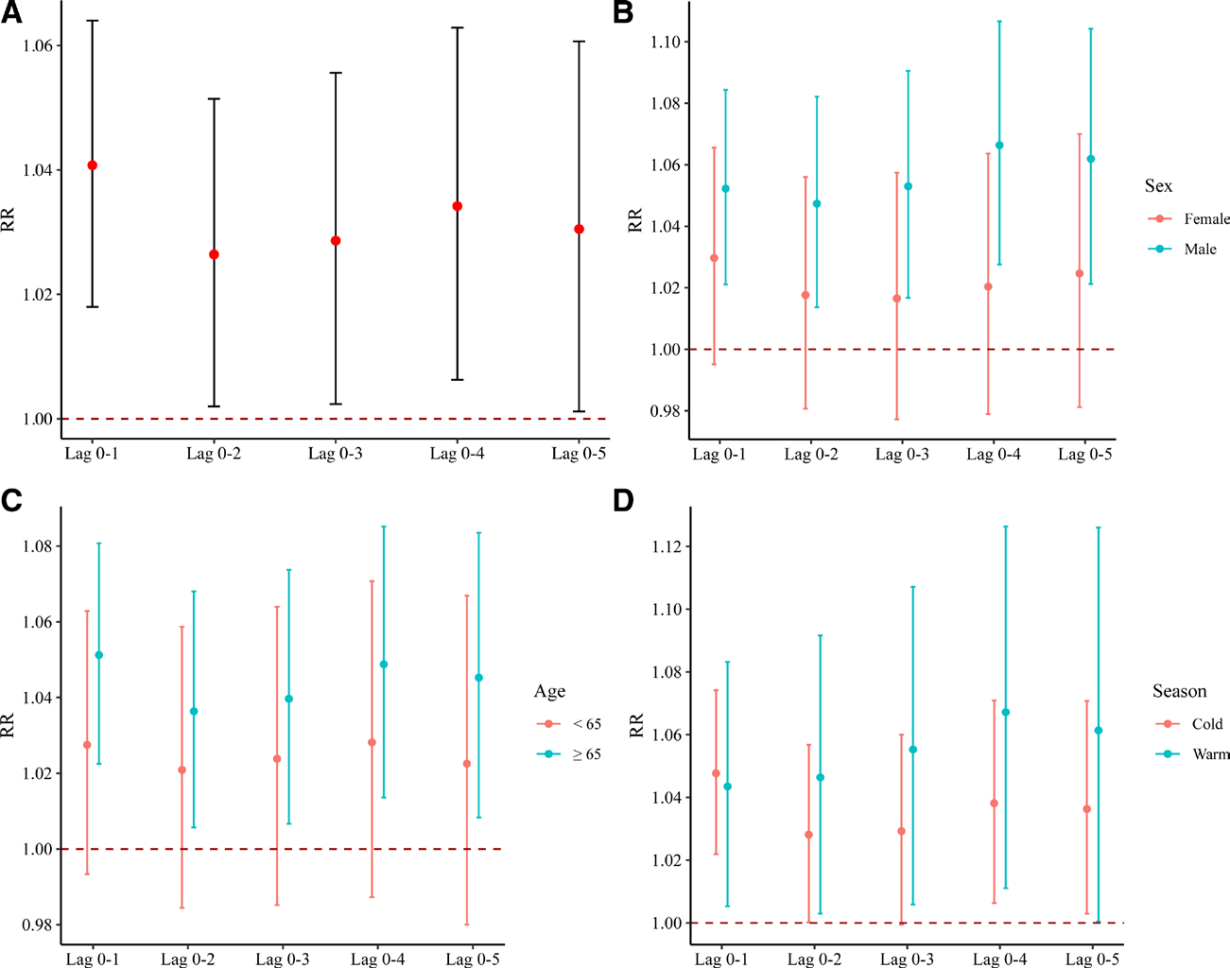
Results for the relationship between each 10 μg/m^3^ increase in SO_2_ and EADs due to CVD (A), and stratified by sex (B), age (C), and season (D) at different cumulative-day lags (lag 0-1 to lag 0-5).

Supplementary analysis of assessing effects on EADs due to CVD at different lag days yielded consistent results with our main findings (Figure 4; STable 2; http://links.lww.com/EE/A301). In general, the strongest effect occurred at lag 0-1 (RR = 1.04, 95% CI: 1.02, 1.06). In single-day lag patterns, the largest effect of SO_2_ with each 10 μg/m^3^ increment on CVD was found at lag 0 (RR= 1.03, 95% CI: 1.02, 1.05) (STable 2; http://links.lww.com/EE/A301). Results from Spearman tests displayed that all other air pollutants were significantly related to SO_2_ concentration (STable 3; http://links.lww.com/EE/A301). Results remained largely similar to our main findings after further adjusting for other air pollutants (STable 4; http://links.lww.com/EE/A301) and adjusted for sex and age (STable 5; http://links.lww.com/EE/A301).

Results of the associations between SO_2_ and EADs due to stroke, MI, HF, and arrhythmia at lag 0-1 are displayed in Figure 2. Each 10 μg/m^3^ increase in SO_2_ was significantly related with EADs due to stroke (RR = 1.05, 95% CI: 1.01, 1.08) and HF (RR = 1.10, 95% CI: 1.03, 1.17). Positive but nonsignificant associations were found for SO_2_ in relation to EADs due to MI (RR = 1.04, 95% CI: 0.96, 1.12) and arrhythmia (RR = 1.05, 95% CI: 0.98, 1.13). Supplementary analysis demonstrated similar results to our main findings (SFigures 4–7; http://links.lww.com/EE/A301). Results of sensitivity analyses remained generally consistent with the main findings after adjusting for sex and age (STable 5; http://links.lww.com/EE/A301). In another sensitivity analysis, the effects of SO_2_ on EADs due to stroke and HF became nonsignificant after adjusting for other air pollutants (STable 6; http://links.lww.com/EE/A301).

## Discussion

In this study, short-term exposure to SO_2_ was found to be significantly associated with increased risk of EADs due to CVD. The relationship between SO_2_ and EADs due to CVD was stronger in males, for participants aged ≥65 years, and in the cold season; however, no significant modification by age, sex, and season was found.

Current evidence of the association between SO_2_ and CVD remained limited and controversial. Our study observed that short-term exposure to SO_2_ was linearly associated with CVD, which was similar to prior studies.^34,35^ However, unlike our findings, another study conducted in England and Wales reported a negative association between SO_2_ and CVD.^12^ The higher concentration of SO_2_ in Guangzhou (median: 11.0 μg/m^3^) than in England and Wales (median: 3.1 μg/m^3^) may help partially interpret this discrepancy. Another reason may rely on the differences in their economic levels and medical resources, in which participants in developed countries could have easier and more access to healthcare for CVD prevention regardless of the differences in air pollutants. Moreover, while previous studies explored the relationship between SO_2_ and CVD in the northern and central regions of China, evidence for the southern areas remained scarce.^32,35,36^ The differences in weather patterns, population susceptibilities, and geographical characteristics between Guangzhou and other regions necessitated further exploration for SO_2_ in relation to CVD risk,^12,37^ especially given that Guangzhou had the highest industrial emissions of SO_2_ among 2407 counties in China.^38^ Therefore, our findings may provide new evidence for the relationship between SO_2_ and CVD and thus may help with strategy development and policy making for CVD prevention.

Some studies have reported potential mechanisms to explain the effect of SO_2_ on CVD.^39–42^ Inhalation of SO_2_ could directly affect the autonomic nervous system, cause a decrease in cardiac vagal control, and induce ventricular arrhythmias.^39,40^ SO_2_ could also substantially interfere with heart rate variability and elevate oxidative stress and inflammation, thereby increasing the risk of CVD.^39,41^

In this study, we used EADs due to CVD as our outcome. It had been argued that EADs could capture accurate participants’ information in real time on acute health events when compared with their electronic medical records.^43^ Indeed, exploring the association between SO_2_ and EADs may help adequately optimize the medical resource allocation for healthcare centers and policy makers especially when the SO_2_ concentration is elevated abruptly. Several previous studies reported positive associations between SO_2_ and EADs due to CVD, which was broadly similar to our findings.^44,45^ A case-crossover study performed in Japan showed that SO_2_ was positively associated with an elevated risk of emergency call for CVD for participants aged ≥65 years.^44^ Our results also indicated a detrimental effect of SO_2_ on participants aged <65 years (Figure 3), highlighting the attention needed to young participants in the public especially when considering the consistently increasing disease burden of CVD in young adults.^46^ Another study conducted in Shanghai reported similar results regarding the increased risk of EADs due to CVD associated with SO_2,_ yet it failed to consider the influence of other air pollutants.^45^ In our sensitivity analysis after adjusting for other air pollutants (STable 4; http://links.lww.com/EE/A301), we also found a significant association between SO_2_ and increased risk of EADs due to CVD, suggesting an independent and detriment effect of SO_2_ on CVD risk.

We primarily assessed the acute effect of SO_2_ at lag 0-1 followed by lag 0-2 to lag 0-5 (Figure 4), in line with other publications.^32,33^ Interestingly, when we performed a post hoc analysis by exploring the cumulative effect of SO_2_ at lag 0-6, a nonsignificant association was found (RR = 1.02, 95% CI: 0.99, 1.05). This implied that the short-term exposure to SO_2_ may have a cumulative lag effect until day 5.

In our analyses, stronger effects were found in males, participants aged ≥65 years, and in the cold season, yet there was no significant modification by subgroup found (Figure 3). The elderly were more sensitive to SO_2_ possibly because of their degradation of organ function, low immune system, and comorbidities.^47^ Higher concentrations of different ambient air pollutants during the cold season might help explain the stronger relationship between SO_2_ and EADs due to CVD in the cold season than warm season.^4^ Unlike other studies,^48,49^ we did not find significant associations between SO_2_ and EADs due to MI and arrhythmia (Figure 2). Additionally, sensitivity analyses demonstrated that the effects of SO_2_ on MI and HF became nonsignificant after further adjusting for other pollutants (STable 6; http://links.lww.com/EE/A301). This inconsistency might be at least in part, due to the small sample size of CVD subcategory events and thus insufficient statistical power. However, results from these subgroup analyses and for secondary outcomes had an exploratory nature and were mainly for hypothesis-generating, requiring interpretation with caution.

This study has several strengths. We used data from the Guangzhou Emergency Center providing a large body of EADs data from various hospitals and communities to clarify the relationship between SO_2_ and CVD risk. Rigorous and detailed analyses also strengthened our study findings. Some limitations should be acknowledged. First, we only had access to EADs data in Guangzhou, which may weaken the generalizability of our findings. Second, due to the lack of data for each air monitoring station, it was not feasible to estimate the pollution exposure levels using finer spatial resolution methods, such as land-use regression models. Data on pollution across all stations were averaged to represent city-level concentration and individual exposure, which might lead to exposure misclassification. However, this approach has been reported to be acceptable in ecological studies.^50^ Furthermore, the majority of EADs cases occurred in the central urban areas of Guangzhou that had over half of the air monitoring stations, which explained the small variability of SO_2_. Third, we could not adjust for other potential confounders (including participants’ education, medication use, lifestyle, and accessibility to medical services, among others), due to the data unavailability. Fourth, because the effect persisted for up to 5 days in this study, using lag 0-1 may underestimate the effect of SO_2_ on EADs due to CVD. Similarly, the relationship between SO_2_ and CVD risk may be underestimated, given the fact that we could not include those participants with acute CVD events who came to hospitals by private vehicles or public transportation.

## Conclusion

To conclude, short-term exposure to SO_2_ was significantly associated with EADs due to CVD. These findings may help adequately optimize the medical resource allocation for healthcare centers and policy makers.

## Conflicts of interest statement

The authors declare that they have no conflicts of interest with regard to the content of this report.

## Supplementary Material

## References

[1] Roth GA Mensah GA Johnson CO; GBD-NHLBI-JACC Global Burden of Cardiovascular Diseases Writing Group. Global burden of cardiovascular diseases and risk factors, 1990-2019: update from the GBD 2019 Study. J Am Coll Cardiol. 2020;76:2982–3021. 33309175 10.1016/j.jacc.2020.11.010 PMC7755038

[2] Tsao CW Aday AW Almarzooq ZI. Heart disease and stroke statistics-2022 update: a report from the American Heart Association. Circulation. 2022;145:e153–e639. 35078371 10.1161/CIR.0000000000001052

[3] Ji JS. Air pollution and cardiovascular disease onset: hours, days, or years? Lancet Public Health. 2022;7:e890–e891. 36334605 10.1016/S2468-2667(22)00257-2

[4] Li X Li Y Yu B. Health and economic impacts of ambient air pollution on hospital admissions for overall and specific cardiovascular diseases in Panzhihua, Southwestern China. J Glob Health. 2022;12:11012. 36538381 10.7189/jogh.12.11012 PMC9805700

[5] Yun Y Hou L Sang N. SO(2) inhalation modulates the expression of pro-inflammatory and pro-apoptotic genes in rat heart and lung. J Hazard Mater. 2011;185:482–488. 20951496 10.1016/j.jhazmat.2010.09.057

[6] Jion M Jannat JN Mia MY. A critical review and prospect of NO(2) and SO(2) pollution over Asia: hotspots, trends, and sources. Sci Total Environ. 2023;876:162851. 36921864 10.1016/j.scitotenv.2023.162851

[7] Wang L Liu C Meng X. Associations between short-term exposure to ambient sulfur dioxide and increased cause-specific mortality in 272 Chinese cities. Environ Int. 2018;117:33–39. 29715611 10.1016/j.envint.2018.04.019

[8] Gong Y Ma R Ren F. Decomposition of industrial SO2 emission in China with firm entry and exit. J Clean Prod. 2023;428:139406.

[9] Al-Kindi SG Brook RD Biswal S Rajagopalan S. Environmental determinants of cardiovascular disease: lessons learned from air pollution. Nat Rev Cardiol. 2020;17:656–672. 32382149 10.1038/s41569-020-0371-2 PMC7492399

[10] Hu X Nie Z Ou Y. Long-term exposure to ambient air pollution, circadian syndrome and cardiovascular disease: a nationwide study in China. Sci Total Environ. 2023;868:161696. 36682545 10.1016/j.scitotenv.2023.161696

[11] Liu Y Guo M Wang J. Effect of short-term exposure to air pollution on hospital admission for cardiovascular disease: a time-series study in Xiangyang, China. Sci Total Environ. 2024;918:170735. 38325454 10.1016/j.scitotenv.2024.170735

[12] Milojevic A Wilkinson P Armstrong B Bhaskaran K Smeeth L Hajat S. Short-term effects of air pollution on a range of cardiovascular events in England and Wales: case-crossover analysis of the MINAP database, hospital admissions and mortality. Heart. 2014;100:1093–1098. 24952943 10.1136/heartjnl-2013-304963 PMC4078678

[13] Le DN Nguyen HAP Ngoc DT. Air pollution and risk of respiratory and cardiovascular hospitalizations in a large city of the Mekong Delta Region. Environ Sci Pollut Res Int. 2022;29:91165–91175. 35881281 10.1007/s11356-022-22022-y

[14] Manca F Lewsey J Mackay D Angus C Fitzpatrick D Fitzgerald N. The effect of a minimum price per unit of alcohol in Scotland on alcohol-related ambulance call-outs: a controlled interrupted time-series analysis. Addiction. 2024;119:846–854. 38286951 10.1111/add.16436

[15] Liu JJ Wang F Liu H. Ambient fine particulate matter is associated with increased emergency ambulance dispatches for psychiatric emergencies. Environ Res. 2019;177:108611. 31401376 10.1016/j.envres.2019.108611

[16] Wang R Tian J Li L. Relationship between diurnal temperature range and emergency ambulance dispatches due to stroke in Guangzhou, China. Sci Total Environ. 2022;817:153037. 35031377 10.1016/j.scitotenv.2022.153037

[17] Hata N Shinada T Kobayashi N. Severity of cardiovascular disease patients transported by air ambulance. Air Med J. 2011;30:328–332. 22055177 10.1016/j.amj.2011.05.004

[18] Thomas TL Clem KJ. Emergency medical services in China. Acad Emerg Med. 1999;6:150–155. 10051908 10.1111/j.1553-2712.1999.tb01054.x

[19] Yang M Wu QZ Zhang YT. Toxicological evaluation and concentration of airborne PM(0.1) in high air pollution period in Guangzhou, China. Sci Total Environ. 2024;921:171224. 38402960 10.1016/j.scitotenv.2024.171224

[20] Wang X Tian J Li Z. Relationship between different particle size fractions and all-cause and cause-specific emergency ambulance dispatches. Environ Health. 2020;19:69. 32552755 10.1186/s12940-020-00619-5 PMC7301562

[21] Yu M Zhu Y Lin CJ. Effects of air pollution control measures on air quality improvement in Guangzhou, China. J Environ Manage. 2019;244:127–137. 31121499 10.1016/j.jenvman.2019.05.046 PMC7652059

[22] Niu Y Chen R Liu C. The association between ambient temperature and out-of-hospital cardiac arrest in Guangzhou, China. Sci Total Environ. 2016;572:114–118. 27497032 10.1016/j.scitotenv.2016.07.205

[23] Lin H Tao J Kan H. Ambient particulate matter air pollution associated with acute respiratory distress syndrome in Guangzhou, China. J Expo Sci Environ Epidemiol. 2018;28:392–399. 29706622 10.1038/s41370-018-0034-0

[24] Yang C Chen X Chen R. Daily ambient temperature and renal colic incidence in Guangzhou, China: a time-series analysis. Int J Biometeorol. 2016;60:1135–1142. 26581758 10.1007/s00484-015-1106-7

[25] Li M Dong H Wang B. Association between ambient ozone pollution and mortality from a spectrum of causes in Guangzhou, China. Sci Total Environ. 2021;754:142110. 32920396 10.1016/j.scitotenv.2020.142110

[26] Gasparrini A Guo Y Hashizume M. Mortality risk attributable to high and low ambient temperature: a multicountry observational study. Lancet. 2015;386:369–375. 26003380 10.1016/S0140-6736(14)62114-0 PMC4521077

[27] Liu R Zeng J Jiang X. The relationship between airborne fine particle matter and emergency ambulance dispatches in a southwestern city in Chengdu, China. Environ Pollut. 2017;229:661–667. 28697471 10.1016/j.envpol.2017.06.098

[28] Sofwan NM Mahiyuddin WRW Latif MT. Risks of exposure to ambient air pollutants on the admission of respiratory and cardiovascular diseases in Kuala Lumpur. Sustain Cities Soc. 2021;75:103390.

[29] Lin H Tao J Qian Z. Shipping pollution emission associated with increased cardiovascular mortality: a time series study in Guangzhou, China. Environ Pollut. 2018;241:862–868. 29913413 10.1016/j.envpol.2018.06.027

[30] Li H Chen R Meng X. Short-term exposure to ambient air pollution and coronary heart disease mortality in 8 Chinese cities. Int J Cardiol. 2015;197:265–270. 26142971 10.1016/j.ijcard.2015.06.050

[31] Chen H Cheng Z Li M. Ambient air pollution and hospitalizations for ischemic stroke: a time series analysis using a distributed lag nonlinear model in Chongqing, China. Front Public Health. 2021;9:762597. 35118040 10.3389/fpubh.2021.762597 PMC8804166

[32] Xu Z Xiong L Jin D Tan J. Association between short-term exposure to sulfur dioxide and carbon monoxide and ischemic heart disease and non-accidental death in Changsha city, China. PLoS One. 2021;16:e0251108. 33939751 10.1371/journal.pone.0251108 PMC8092655

[33] Zhang C Ding R Xiao C. Association between air pollution and cardiovascular mortality in Hefei, China: a time-series analysis. Environ Pollut. 2017;229:790–797. 28797522 10.1016/j.envpol.2017.06.022

[34] Liu M Yu J Zhu A. Association between air pollution and coronary heart disease hospitalizations in Lanzhou City, 2013-2020: a time series analysis. J Urban Health. 2023;100:1246–1257. 38010484 10.1007/s11524-023-00797-w PMC10728394

[35] Amsalu E Guo Y Li H. Short-term effect of ambient sulfur dioxide (SO2) on cause-specific cardiovascular hospital admission in Beijing, China: a time series study. Atmos Environ. 2019;208:74–81.

[36] Venners SA Wang B Xu Z Schlatter Y Wang L Xu X. Particulate matter, sulfur dioxide, and daily mortality in Chongqing, China. Environ Health Perspect. 2003;111:562–567. 12676616 10.1289/ehp.5664 PMC1241445

[37] Chen L Wang X Qian ZM. Ambient gaseous pollutants and emergency ambulance calls for all-cause and cause-specific diseases in China: a multicity time-series study. Environ Sci Pollut Res Int. 2022;29:28527–28537. 34988821 10.1007/s11356-021-18337-x

[38] Jiang C Pei C Cheng C. Emission factors and source profiles of volatile organic compounds from typical industrial sources in Guangzhou, China. Sci Total Environ. 2023;869:161758. 36702262 10.1016/j.scitotenv.2023.161758

[39] Routledge HC Manney S Harrison RM Ayres JG Townend JN. Effect of inhaled sulphur dioxide and carbon particles on heart rate variability and markers of inflammation and coagulation in human subjects. Heart. 2006;92:220–227. 15923279 10.1136/hrt.2004.051672 PMC1860755

[40] Tunnicliffe WS Hilton MF Harrison RM Ayres JG. The effect of sulphur dioxide exposure on indices of heart rate variability in normal and asthmatic adults. Eur Respir J. 2001;17:604–608. 11401052 10.1183/09031936.01.17406040

[41] Chuang KJ Chan CC Su TC Lee CT Tang CS. The effect of urban air pollution on inflammation, oxidative stress, coagulation, and autonomic dysfunction in young adults. Am J Respir Crit Care Med. 2007;176:370–376. 17463411 10.1164/rccm.200611-1627OC

[42] Zhu M Du J Liu AD Holmberg L Tang C Jin H. Effect of endogenous sulfur dioxide in regulating cardiovascular oxidative stress. Histol Histopathol. 2014;29:1107–1111. 24718903 10.14670/HH-29.1107

[43] Cheng J Xu Z Zhao D. The burden of extreme heat and heatwave on emergency ambulance dispatches: a time-series study in Huainan, China. Sci Total Environ. 2016;571:27–33. 27454572 10.1016/j.scitotenv.2016.07.103

[44] Yorifuji T Suzuki E Kashima S. Cardiovascular emergency hospital visits and hourly changes in air pollution. Stroke. 2014;45:1264–1268. 24692477 10.1161/STROKEAHA.114.005227

[45] Jiang J Wu D Chen Y Han Y Jin W. Relationship between different air pollutants and total and cause-specific emergency ambulance dispatches in Shanghai, China. Int Arch Occup Environ Health. 2021;94:1709–1719. 34319408 10.1007/s00420-021-01743-6

[46] Tong Z Xie Y Li K Yuan R Zhang L. The global burden and risk factors of cardiovascular diseases in adolescent and young adults, 1990-2019. BMC Public Health. 2024;24:1017. 38609901 10.1186/s12889-024-18445-6 PMC11010320

[47] Chen TT Zhan ZY Yu YM Xu L-J Guan Y Ou C-Q. Effects of hourly levels of ambient air pollution on ambulance emergency call-outs in Shenzhen, China. Environ Sci Pollut Res Int. 2020;27:24880–24888. 32337675 10.1007/s11356-020-08416-w

[48] Xue X Hu J Xiang D. Hourly air pollution exposure and the onset of symptomatic arrhythmia: an individual-level case-crossover study in 322 Chinese cities. CMAJ. 2023;195:E601–E611. 37127306 10.1503/cmaj.220929 PMC10151095

[49] Chen R Jiang Y Hu J. Hourly air pollutants and acute coronary syndrome onset in 1.29 million patients. Circulation. 2022;145:1749–1760. 35450432 10.1161/CIRCULATIONAHA.121.057179

[50] Kim D Sass-Kortsak A Purdham JT Dales RE Brook JR. Associations between personal exposures and fixed-site ambient measurements of fine particulate matter, nitrogen dioxide, and carbon monoxide in Toronto, Canada. J Expo Sci Environ Epidemiol. 2006;16:172–183. 16175198 10.1038/sj.jea.7500446

