# Supplementary material for: Relationship between short-term exposure to sulfur dioxide and emergency ambulance dispatches due to cardiovascular disease
Source: Environ Epidemiol. 2024 Sep 24;8(5):e341. doi: 10.1097/EE9.0000000000000341 (PMC11424135; doi:10.1097/EE9.0000000000000341)
Supplement: Supplementary file 1 [file ee9-8-e341-s001.pdf]

Supplemental data

List of contents:

SFigure 1. The geographic location of air monitoring stations in Guangzhou, China ..2

SFigure 2. Distribution of SO_2 concentration between October 2013 and June 2018 in Guangzhou...3

SFigure 3. The time series of daily numbers of EADs between October 2013 and June 2018 in Guangzhou 4

SFigure 4. Results for the relationship between each 10 μg/m^3 increase in SO_2 and EADs due to stroke at different cumulative-day lags (lag 0-1 to lag 0-5) 5

SFigure 5. Results for the relationship between each 10 μg/m^3 increase in SO_2 and EADs due to MI at cumulative-day lags (lag 0-1 to lag 0-5) 6

SFigure 6. Results for the relationship between each 10 μg/m3 increase in SO2 and EADs due to HF at different cumulative-day lags (lag 0-1 to lag 0-5) 7

SFigure 7. Results for the relationship between each 10 μg/m^3 increase in SO_2 and EADs due to arrhythmia at different cumulative-day lags (lag 0-1 to lag 0-5) 8

STable 1. Measures of Q-AIC by different df for time trend and meteorological variables in the models* 9

STable 2. Results for the relationship between each 10 μg/m^3 increase in SO_2 and EADs due to CVD at different single-day lags (lag 0 to lag 5) 10

STable 3. Correlations between the daily average concentration of SO_2 and other air pollutants 11

STable 4. Sensitivity analysis for EADs due to CVD associated with each 10 μg/m^3 increase in SO_2 at lag 0-1 after further adjusted for $PM_{2.5}$, PM_{10}, O_3, NO_2, and CO* 12

STable 5. Sensitivity analysis for EADs due to CVD, stroke, MI, HF, and arrhythmia associated with each 10 μg/m^3 increase in SO_2 at lag 0-1 after further adjusted for sex and age* 13

STable 6. Sensitivity analysis for EADs due to stroke, MI, HF, and arrhythmia associated with each 10 μg/m^3 increase in SO_2 at lag 0-1 after further adjusted for $PM_{2.5}$, PM_{10}, O_3, NO_2, and CO*..14

SFigure 1. The geographic location of air monitoring stations in Guangzhou, China

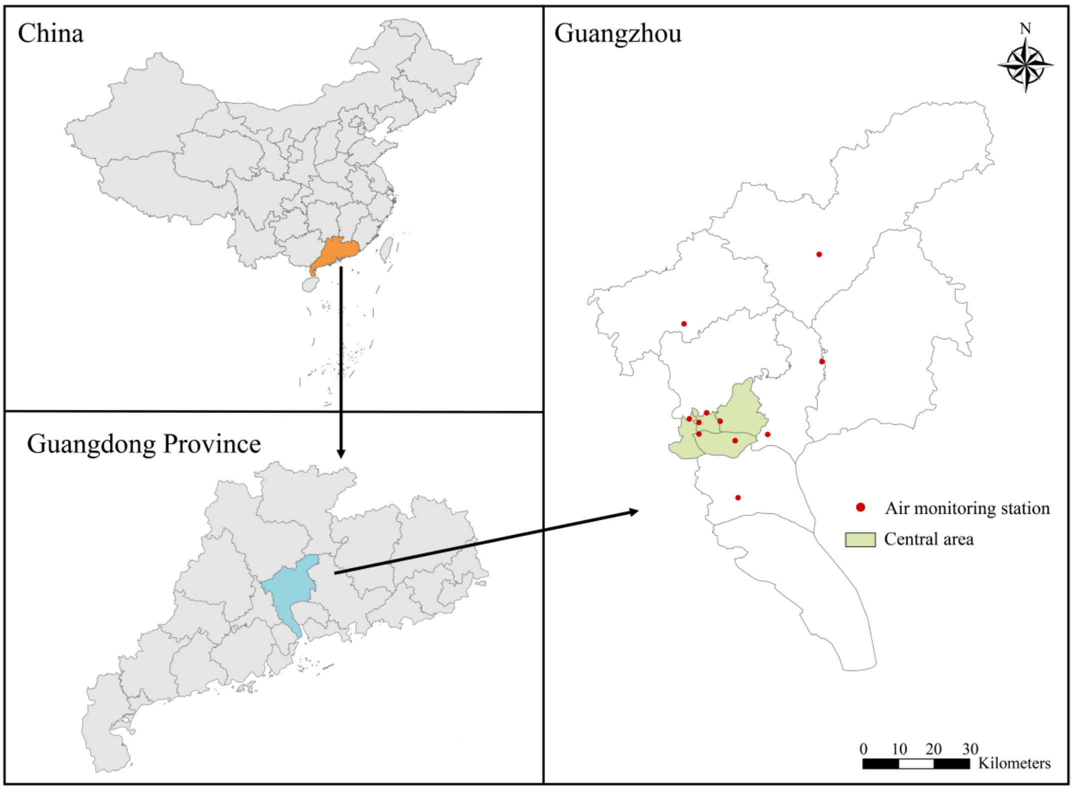

SFigure 2. Distribution of SO_2 concentration between October 2013 and June 2018 in Guangzhou

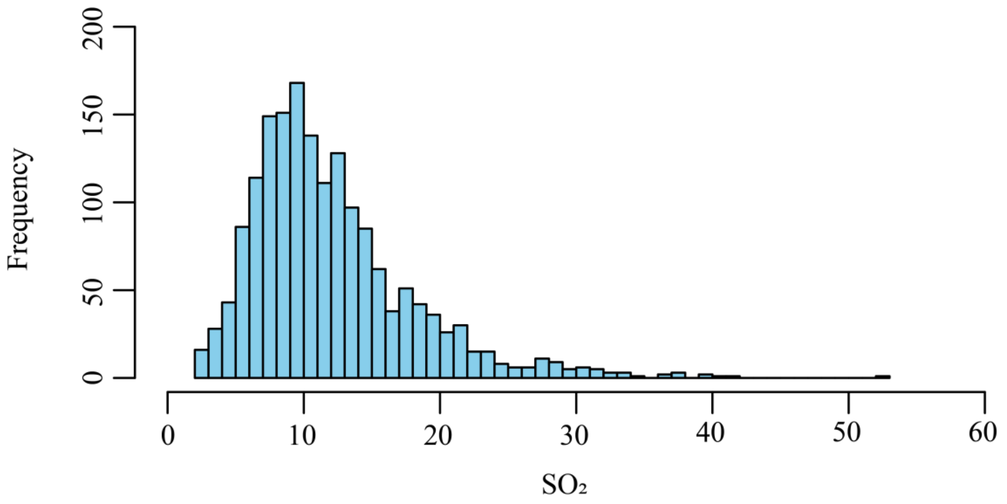

Note: SO_2 = sulfur dioxide.

SFigure 3. The time series of daily numbers of EADs between October 2013 and June 2018 in Guangzhou

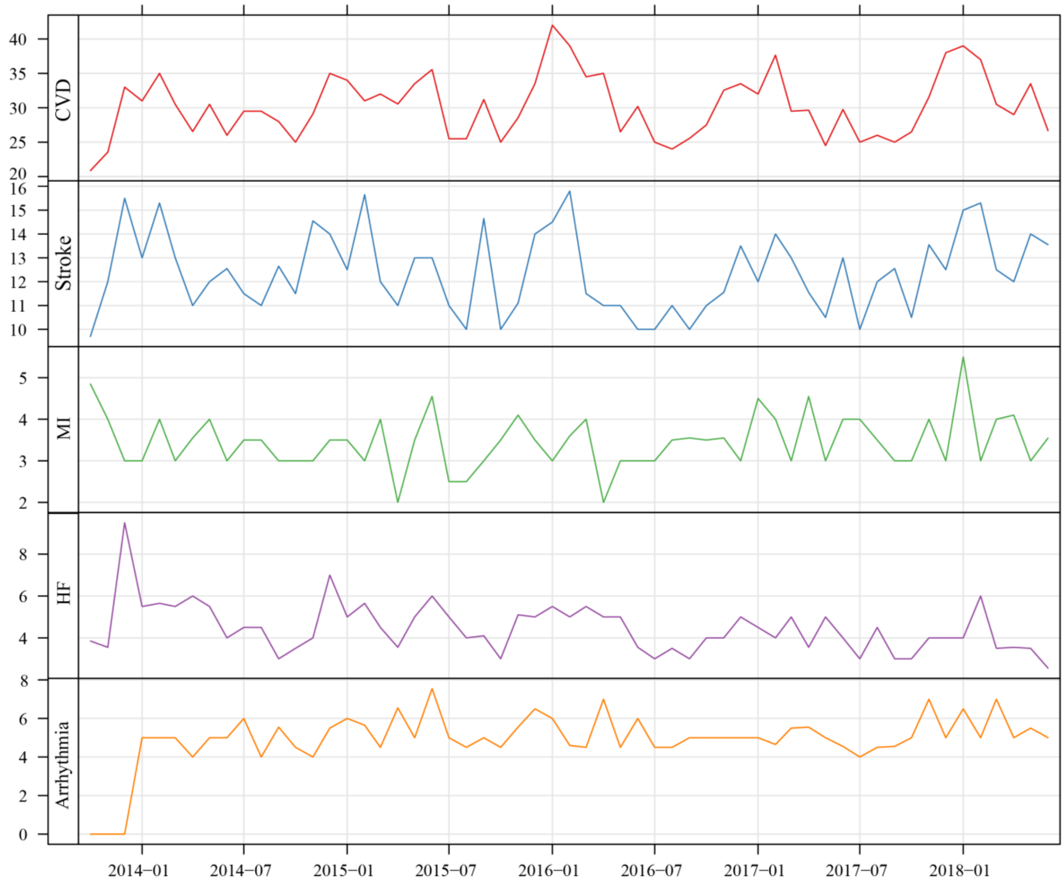

Note: EADs = emergency ambulance dispatches; CVD = cardiovascular disease; MI = myocardial infarction; HF = heart failure.

SFigure 4. Results for the relationship between each 10 μg/m^3 increase in SO_2 and EADs due to stroke at different cumulative-day lags (lag 0-1 to lag 0-5)

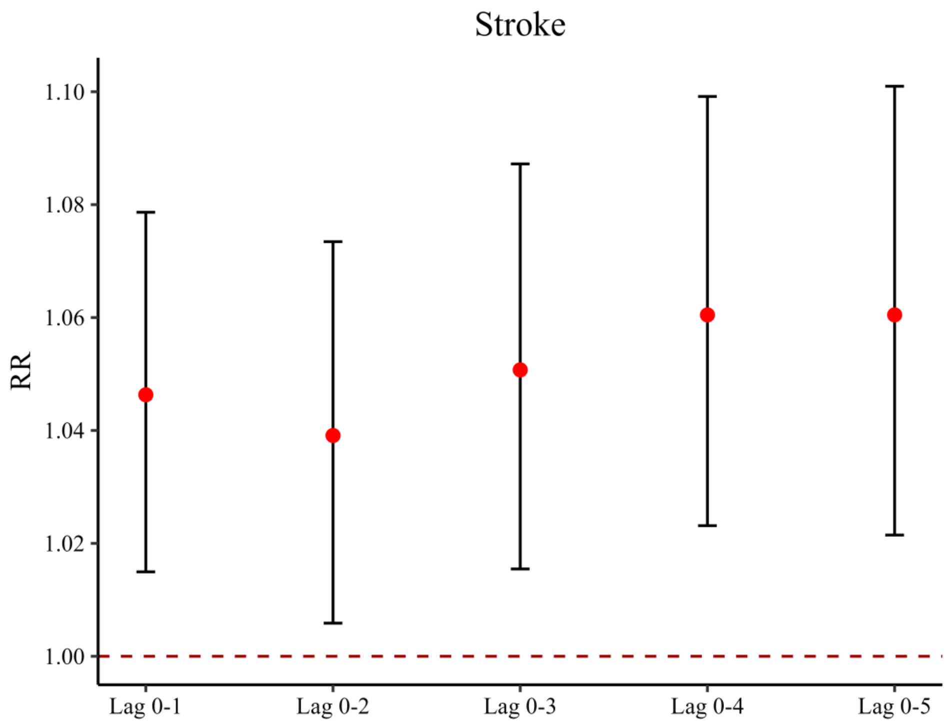

Note: RR = relative risk; EADs = emergency ambulance dispatches; SO_2 = sulfur dioxide.

SFigure 5. Results for the relationship between each 10 μg/m^3 increase in SO_2 and EADs due to MI at cumulative-day lags (lag 0-1 to lag 0-5)

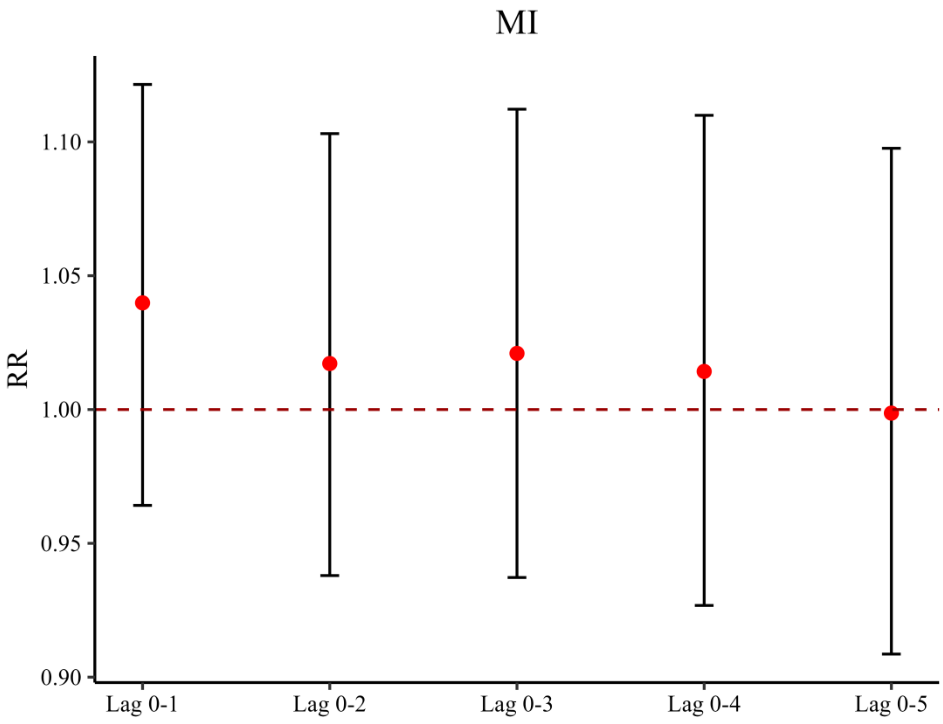

Note: RR = relative risk; EADs = emergency ambulance dispatches; MI = myocardial infarction; SO_2 = sulfur dioxide.

SFigure 6. Results for the relationship between each 10 μg/m3 increase in SO2 and EADs due to HF at different cumulative-day lags (lag 0-1 to lag 0-5)

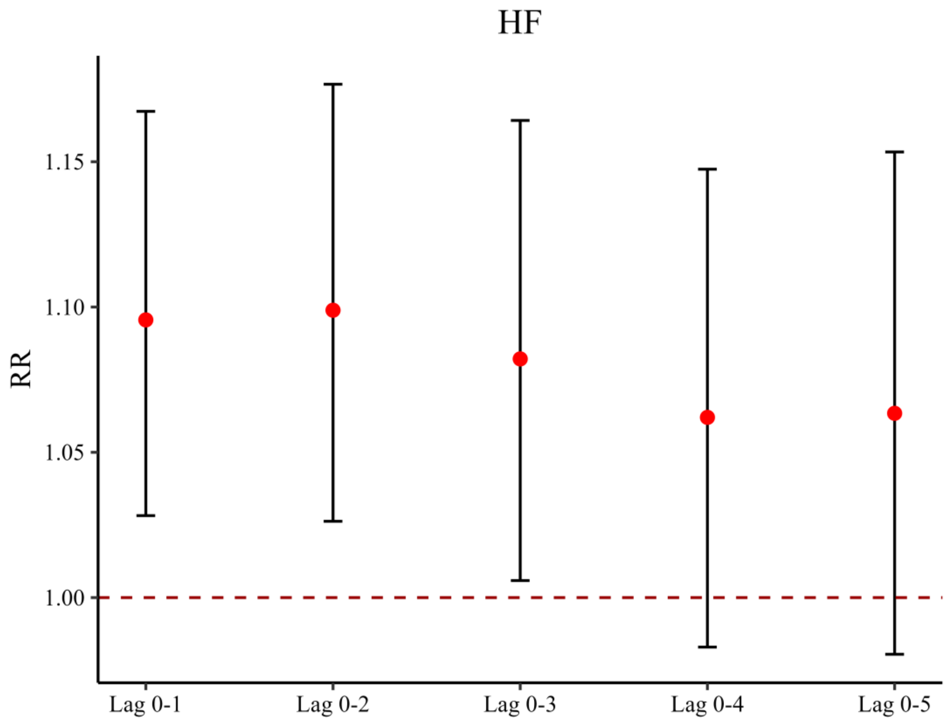

Note: RR = relative risk; CI = confidence interval; EADs = emergency ambulance dispatches; HF = heart failure; SO_2 = sulfur dioxide.

SFigure 7. Results for the relationship between each 10 μg/m^3 increase in SO_2 and EADs due to arrhythmia at different cumulative-day lags (lag 0-1 to lag 0-5)

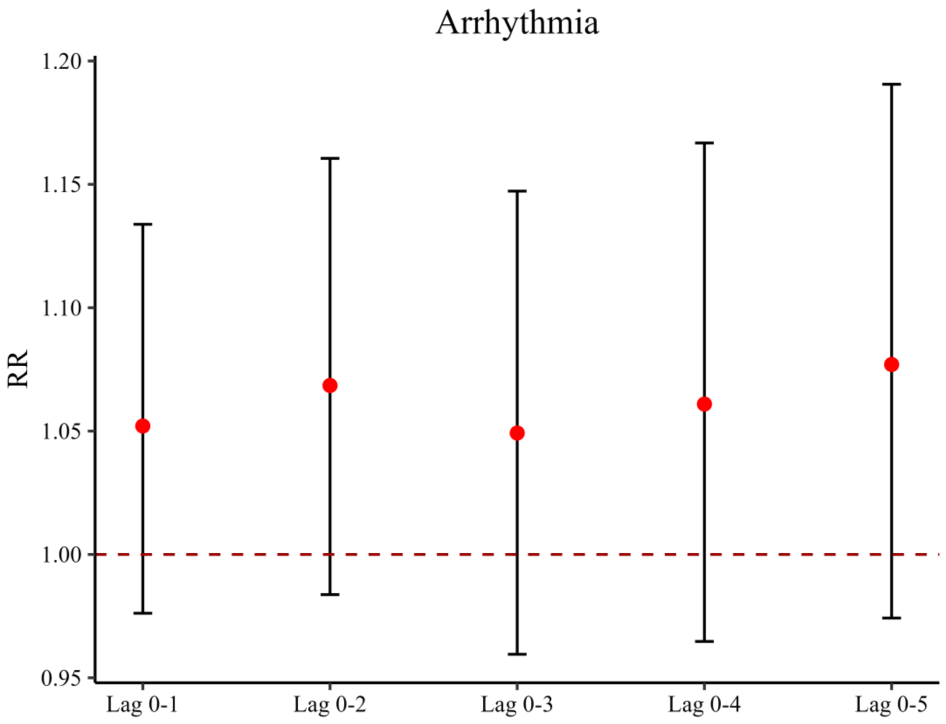

Note: RR = relative risk; CI = confidence interval; EADs = emergency ambulance dispatches; SO_2 = sulfur dioxide.

STable 1. Measures of Q-AIC by different df for time trend and meteorological variables in the models*

df for temperature	df for relative humidity	df for time trend		
		df = 6	df = 7	df = 8
df = 3				
	df = 3	**10330.38**[#]	10345.12	10348.55
	df = 4	10332.65	10347.33	10350.81
	df = 5	10335.07	10349.74	10353.21
df = 4				
	df = 3	10332.50	10347.27	10350.75
	df = 4	10334.77	10349.47	10353.01
	df = 5	10337.19	10351.89	10355.41
df = 5				
	df = 3	10334.99	10349.68	10353.15
	df = 4	10337.28	10351.90	10355.42
	df = 5	10339.69	10354.32	10357.82

Q-AIC = Quasi-Akaike Information Criterion; df = degree of freedom.

* Previous studies reported that df for time trend ranged from 6 to 8, and df for meteorological variables ranged from 3 to 5.

[#] This Q-AIC value was the smallest among all the measures.

STable 2. Results for the relationship between each 10 μg/m^3 increase in SO_2 and EADs due to CVD at different single-day lags (lag 0 to lag 5)

**Outcomes**	**Lag 0**	**Lag 1**	**Lag 2**	**Lag 3**	**Lag 4**	**Lag 5**
CVD	1.03 (1.02, 1.05)	1.01 (1.00, 1.02)	0.99 (0.98, 1.00)	0.99 (0.98, 1.00)	1.00 (0.99, 1.00)	1.01 (0.99, 1.02)
By sex						
Male	1.04 (1.02, 1.07)	1.01 (1.00, 1.03)	0.99 (0.98, 1.01)	0.99 (0.98, 1.00)	1.00 (0.99, 1.01)	1.02 (0.99, 1.04)
Female	1.03 (0.99, 1.05)	1.01 (0.99, 1.02)	0.99 (0.98, 1.01)	0.99 (0.98, 1.01)	0.99 (0.99, 1.01)	1.01 (0.98, 1.03)
By age						
< 65	1.02 (0.99, 1.05)	1.01 (0.99, 1.02)	1.00 (0.98, 1.00)	0.99 (0.98, 1.01)	0.99 (0.99, 1.01)	1.00 (0.97, 1.03)
≥ 65	1.04 (1.02, 1.06)	1.01 (1.00, 1.02)	0.99 (0.98, 1.01)	0.99 (0.98, 1.00)	0.99 (0.99, 1.01)	1.01 (0.99, 1.03)
By season						
Warm	1.02 (0.99, 1.05)	1.01 (1.00, 1.03)	1.01 (0.99, 1.02)	1.00 (0.99, 1.02)	1.00 (0.99, 1.02)	1.01 (0.98, 1.03)
Cold	1.04 (1.02, 1.07)	1.01 (1.00, 1.02)	0.99 (0.98, 1.00)	0.98 (0.97, 0.99)	0.99 (0.99, 1.03)	1.01 (0.99, 1.03)

STable 3. Correlations between the daily average concentration of SO_2 and other air pollutants

**Air pollutants**	**SO_2**	**$PM_{2.5}$**	**PM_{10}**	**NO_2**	**O_3**	**CO**
SO_2	1.00	-	-	-	-	-
$PM_{2.5}$	0.71*	1.00	-	-	-	-
PM_{10}	0.75*	0.97*	1.00	-	-	-
NO_2	0.59*	0.73	0.75*	1.00	-	-
O_3	0.29*	0.30*	0.35*	-0.01*	1.00	-
CO	0.37*	0.61*	0.55*	0.57*	-0.20*	1.00

SO_2 = sulfur dioxide; $PM_{2.5}$ = particulate matter less than 2.5 μm in aerodynamic diameter; PM_{10} = particulate matter less than 10 μm in aerodynamic diameter; NO_2 = nitrogen dioxide; O_3 = ozone; CO = carbon monoxide.

$^*P < 0.05$

STable 4. Sensitivity analysis for EADs due to CVD associated with each 10 μg/m^3 increase in SO_2 at lag 0-1 after further adjusted for $PM_{2.5}$, PM_{10}, O_3, NO_2, and CO*

**Outcomes**	**RR (95% CI)**	***P*-value**
CVD	1.04 (1.01, 1.07)	0.04
By sex		
Male	1.03 (1.00, 1.10)	0.04
Female	1.02 (0.97, 1.07)	0.77
By age		
< 65	1.02 (0.97, 1.07)	0.64
≥ 65	1.04 (1.01, 1.09)	0.04
By season		
Warm	1.14 (0.98, 1.33)	0.11
Cold	1.03 (0.99, 1.07)	0.10

EADs = emergency ambulance dispatches; CVD = cardiovascular disease; SO_2 = sulfur dioxide; $PM_{2.5}$ = particulate matter less than 2.5 μm in aerodynamic diameter; PM_{10} = particulate matter less than 10 μm in aerodynamic diameter; NO_2 = nitrogen dioxide; O_3 = ozone; RR = relative risk; CI = confidence interval.

* Model adjusted for $PM_{2.5}$, PM_{10}, O_3, NO_2, and CO, and long-term trend, mean temperature, relative humidity, day of week and public holidays.

STable 5. Sensitivity analysis for EADs due to CVD, stroke, MI, HF, and arrhythmia associated with each 10 μg/m^3 increase in SO_2 at lag 0-1 after further adjusted for sex and age*

**Outcomes**	**RR (95% CI)**	***P*-value**
CVD	1.04 (1.02, 1.06)	< 0.01
Stroke	1.04 (1.01, 1.07)	< 0.01
MI	1.04 (0.99, 1.08)	0.28
HF	1.05 (1.01, 1.10)	0.03
Arrhythmia	1.05 (0.98, 1.13)	0.41

EADs = emergency ambulance dispatches; CVD = cardiovascular disease; MI = myocardial infarction; HF = heart failure; SO_2 = sulfur dioxide; RR = relative risk; CI = confidence interval.

* Model adjusted for sex, age, long-term trend, mean temperature, relative humidity, day of week and public holidays.

STable 6. Sensitivity analysis for EADs due to stroke, MI, HF, and arrhythmia associated with each 10 μg/m^3 increase in SO_2 at lag 0-1 after further adjusted for $PM_{2.5}$, PM_{10}, O_3, NO_2, and CO*

Outcomes	RR (95% CI)	*P*-value
Stroke	1.04 (0.99, 1.09)	0.25
MI	1.02 (0.91, 1.15)	0.57
HF	1.06 (0.97, 1.16)	0.40
Arrhythmia	1.06 (0.97, 1.16)	0.87

EADs = emergency ambulance dispatches; MI = myocardial infarction; HF = heart failure; SO_2 = sulfur dioxide; $PM_{2.5}$ = particulate matter less than 2.5 μm in aerodynamic diameter; PM_{10} = particulate matter less than 10 μm in aerodynamic diameter; NO_2 = nitrogen dioxide; O_3 = ozone; RR = relative risk; CI = confidence interval.

* Model adjusted for $PM_{2.5}$, PM_{10}, O_3, NO_2, and CO, and long-term trend, mean temperature, relative humidity, day of week and public holidays.
